# Assessment of Surgeons’ Stress Levels with Digital Sensors during Robot-Assisted Surgery: An Experimental Study

**DOI:** 10.3390/s24092915

**Published:** 2024-05-02

**Authors:** Kristóf Takács, Eszter Lukács, Renáta Levendovics, Damján Pekli, Attila Szijártó, Tamás Haidegger

**Affiliations:** 1 Antal Bejczy Center for Intelligent Robotics (IROB), University Research and Innovation Center (EKIK), Óbuda University, 1034 Budapest, Hungary; eszter.lukacs@irob.uni-obuda.hu (E.L.); renata.levendovics@irob.uni-obuda.hu (R.L.); 2 John von Neumann Faculty of Informatics (NIK), Óbuda University, 1034 Budapest, Hungary; 3 Austrian Center for Medical Innovation and Technology (ACMIT), 2700 Wiener Neustadt, Austria; 4 Department of Surgery, Transplantation and Gastroenterology, Semmelweis University, 1082 Budapest, Hungary; damjan.pkli@gmail.com (D.P.); szijartoatila@gmail.com (A.S.)

**Keywords:** robot-assisted minimally invasive surgery, human–robot cooperation, stress analysis, non-technical surgical skills, clinical situation awareness

## Abstract

Robot-Assisted Minimally Invasive Surgery (RAMIS) marks a paradigm shift in surgical procedures, enhancing precision and ergonomics. Concurrently it introduces complex stress dynamics and ergonomic challenges regarding the human–robot interface and interaction. This study explores the stress-related aspects of RAMIS, using the da Vinci XI Surgical System and the Sea Spikes model as a standard skill training phantom to establish a link between technological advancement and human factors in RAMIS environments. By employing different physiological and kinematic sensors for heart rate variability, hand movement tracking, and posture analysis, this research aims to develop a framework for quantifying the stress and ergonomic loads applied to surgeons. Preliminary findings reveal significant correlations between stress levels and several of the skill-related metrics measured by external sensors or the SURG-TLX questionnaire. Furthermore, early analysis of this preliminary dataset suggests the potential benefits of applying machine learning for surgeon skill classification and stress analysis. This paper presents the initial findings, identified correlations, and the lessons learned from the clinical setup, aiming to lay down the cornerstones for wider studies in the fields of clinical situation awareness and attention computing.

## 1. Background

### 1.1. Robot-Assisted Surgery

Robotic assistance may provide healthcare support to both patients and caregivers at various levels. Robotic surgery represents a significant advancement in the field of Minimally Invasive Surgery (MIS), with the da Vinci surgical robot being the prime example [1], having seen five generations of evolving telerobotic platforms. While the principle of Robot-Assisted Minimally Invasive Surgery (RAMIS) relies on tele-operation, these complex systems are considered to be efficient clinical robots [2]. Human–robot interaction technology allows surgeons to perform complex procedures with increased precision, flexibility, and control compared to traditional techniques, while new devices such as autonomous surgical systems [3,4], technologies such as medical 3D printing [5], and supporting systems such as robot assistance [6] are being continuously developed by the research community. Today, Artificial Intelligence (AI) and Machine Learning (ML) methods are opening up new frontiers in robotic surgery [1], while regulatory bodies are barely able to keep pace [7].

Human–robot cooperation is primarily maintained by the da Vinci surgical system through a console where the surgeon operates by applying controls and a robotic platform executes the motions with high precision [8,9]. While RAMIS is sometimes considered to be a costly technological add-on to surgery, although one preferred by patients [10], it has also been seen as an initial component in the move towards sustainable and accessible healthcare [9]. More recently, RAMIS has been presented as a general means to support ethically aligned design in digital health devices [11].

Despite advancements, the Operating Room (OR) remains a high-stress environment, characterized by tasks requiring precise and coordinated actions [12]. In RAMIS, the surgeon has no direct physical interaction with the patient, allowing them to focus on the ergonomic and psychological aspects of surgery instead of on hygiene, as the surgeon’s close environment is less regulated in this new scenario. However, because full control of the surgical procedure and all decision-making remains the surgeon’s burden, the effect of human error remains one of the biggest concerns in RAMIS. Errors can originate from a variety of factors, including stress, fatigue, and the complexity of the procedures themselves [13,14]. Recognizing and addressing these errors is crucial, as they can lead to complications, extended recovery times, and in some cases even irreversible damage.

Computer-integrated surgery has seen significant technical challenges on the top of the clinical complications [15,16]. Recognizing adverse events in a sufficient time remains key to preventing any negative patient outcome, which requires the maintenance of focus and situational awareness on the part of both the surgeon and the OR team [17,18].

### 1.2. Ergonomics and Stress in Surgery

While assistive systems such as the da Vinci robot primarily reduce physical strain through enhanced ergonomics, cognitive and emotional stress remains significant. This is further emphasized by the unconventional setup by which surgeons indirectly control a whole robotic system.

Nonetheless, a notable advantage of RAMIS over traditional open (or laparoscopic) surgery is the improved ergonomics. Unlike laparoscopic surgery, which requires surgeons to maintain unnatural and uncomfortable positions, robotic surgery consoles generally allow better posture and arm support while enabling surgeons to stand up and stretch, as the robot can keep the tools in a stable position for an unlimited time. This ergonomic setup reduces physical fatigue, potentially enhances performance, and reduces the probability of long-term musculoskeletal disorders [19].

Understanding stress patterns and ergonomic challenges allows surgeons to modify techniques, take breaks, and adjust the OR setup, thereby optimizing their performance. Real-time stress monitoring can prompt breaks in the process or allow for modifications and adjustments, thereby improving decision-making and surgical precision, while ergonomic feedback can guide adjustments in console settings or posture, preventing long-term physical strain.

In this paper an innovative approach for a novel OR stress and ergonomics inspection framework is proposed. Although each sensor component of the system (hand and posture tracking, electrocardiography, SURG-TLX, skill level classification, etc.) has already been introduced to RAMIS, the novelty of this research lies in the interconnection of these methods. The authors believe that the presented correlations and lack of correlations in the recorded dataset along with the identified gaps and corresponding future plans for this experiment will offer a valuable basis for research activities related to non-technical skills in RAMIS, thereby improving circumstances in the OR in the long term.

We present the design, implemented setup, and first stage of the in vitro experiment in Section 2 (Methods), the preliminary results in Section 3 (Results), and our conclusions and lessons learned in Section 4 (Conclusions).

## 2. Methods

Measuring stress levels and OR ergonomics is vital for understanding their impact on surgeons’ mental and physical well-being. Current stress estimation methods vary from physiological measures such as Heart Rate Variability (HRV), eye movements, and cortisol levels to psychological means such as self-reported stress-measuring questionnaires. Recent technologies, including wearable sensors and machine learning algorithms, offer even more advanced stress assessment based on numerous different data inputs [20].

**Figure 1:**
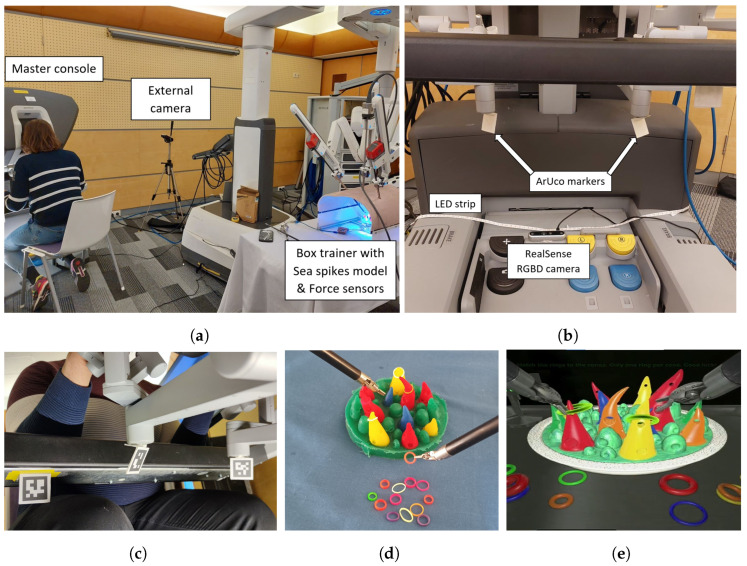
Experimental setup. The subject is sitting at the master console of the da Vinci XI robot while her/his posture is recorded by an external camera from the side (**a**). The sea spikes model (**d**) is placed in a box trainer onto force sensors (**a**). The positions of the MTMs are recorded by tracking the attached ArUco markers (**c**) using a RealSense D455 RGB-D camera (**b**). The simulated sea spikes model (**e**) was used in the da Vinci Skill Simulator as well.

Ergonomics in RAMIS can be evaluated using biomechanical analysis, motion capture, and pressure mapping. These methods help to identify factors related to ergonomics in the OR contributing to physical strain and inefficiency on the part of surgeons, both of which can negatively impact surgical outcomes [19,21]. Figure 1 shows the chosen equipment and the experimental setup of this study.

### 2.1. Sensors and Measurements

The main goal of our experiment was to assess and quantify surgeons’ workload and stress levels when using RAMIS and to examine any correlations between these and other more commonly measured metrics. The recorded data were used for skill level distinction as well. The most important tool for stress assessment was a Polar H10 heart rate sensor band (Polar Electro OY, headquarters: Kempele, Finland) with the Polar Android application. In addition, the hand movements and posture of the subjects were recorded and self-assessment questionnaires were filled out before and after the trials.

#### 2.1.1. Heart Rate Measurement

The stress level of the subjects was estimated using the Baevsky Stress Index (BSI), proposed by Baevsky in [22]. The BSI can be calculated from a time series of RR-intervals (the time elapsed in ms between two successive R-waves on the electrocardiogram, i.e., the reciprocate of the heart rate) recorded by any heart rate measuring device with the following formula:

(1) $$SI=\frac{AM_{o}\times 100\%}{2M_{o}\times M_{x}DM_{n}}.$$

Equation (1) uses the RR interval data rounded to 50 ms for noise reduction; $M_{0}$ denotes the mode and ${\mathrm{AM}}_{0}$ is the amplitude of the mode, i.e., the frequency of occurrence of the mode in the whole dataset in percentage, while $M_{x}DM_{n}$ denotes the difference between the longest and the shortest RR-interval values. This formula utilizes Heart Rate Variability (HRV) analysis to estimate the stress level, which is a widely used approach in many fields of medicine [22,23].

#### 2.1.2. Posture Detection

**Figure 2:**
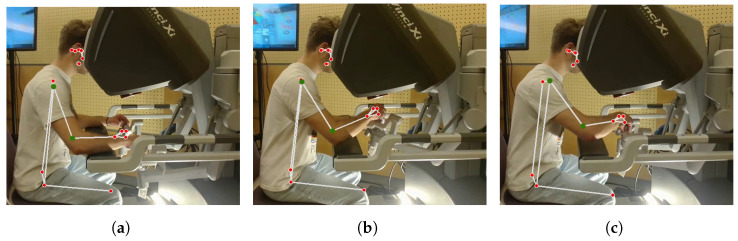
Posture detection visualization in different arm positions. The red circles denote the anatomical landmarks within the human body that are identifiable through the Python code, while the green circles specifically represent the tracked points corresponding to the right shoulder and right elbow. Figures (**a**–**c**) exhibit three posture of the same participant in one trial, as typical examples.

Posture detection involved the utilization of a custom Python code designed to identify 33 specific points within the human body, represented as red circles in Figure 2.The code execution was not performed in real-time; instead, an external camera on the participants’ right side was used for recording the trials. In this way, the recorded videos enabled synchronization between heart rate measurements, hand movements, and posture data. Due to the position of the external camera, not all of the 33 landmarks can be seen in the recorded videos. The camera was positioned to only capture the participants’ bodies from the knees to the head. Therefore, the points that could have been detected in the lower leg parts and the points that were covered by the machine were missing. This camera configuration was a consequence of the fact that the posture detection algorithm was narrowed down to the identification of key points, denoted by green circles in Figure 2.

#### 2.1.3. Hand Movement Tracking

Hand movements, i.e., the movements of the master tool manipulators (MTMs), were tracked using an external RealSense D455 camera (Intel Co., Santa Clara, CA, USA) and ArUco markers (Figure 1). It was important that the clinical da Vinci remain completely intact; thus, the markers were merely glued onto the MTMs with 3D printed holders, and an additional marker was affixed to the bottom of the armrest to provide a stable reference. Thanks to the mechanical design of the MTMs, the markers can be attached at the position shown in Figure 1 for position tracking, as the rest of the joints (the three joints between the marker and the hand) only control the orientation. In this way, the position of the MTMs could be tracked with one common camera and one marker each, and would not be covered by the hands or parts of the MTMs; on the other hand, the orientation information is lost [24].

The spatial positions of the markers were tracked and saved in semi-real-time at 10 Hz using a Python script (with the ArUco library of OpenCV) running on a standard Windows PC (code developed by the authors in Python v3.8). The ArUco library provided a list of the detected markers on the video frames, each marker was represented by its rotation(rvec) and translation (tvec) vectors. To obtain an absolute position for the markers attached to the MTMs, their positions needed to be transformed into the coordinate system of the fixed marker, as the camera might have been only loosely attached to the console (see Figure 1).

First, the *z* component (i.e., distance from the camera, calculated by the ArUco library using the known size of the marker) for each marker’s position vector was replaced with the more accurate depth value of the RGB-D camera. Then, the *rvec* and *tvec* vectors were transformed into homogeneous matrices using the Rodrigues formula [25]. Using the homogeneous matrix representations of the ArUco markers, the spatial positions of the two moving markers ($H_{2}$ and $H_{3}$) were transformed into the fixed marker’s coordinate system ($H_{1}$):

(2) $$\begin{array}{cccc} H_{2} & =\left[\begin{array}{cc} \mathrm{Rodriguez}(rvec_{2}) & tvec_{2}\\ 0 & 1 \end{array}\right], & \;H_{3} & =\left[\begin{array}{cc} \mathrm{Rodriguez}(rvec_{3}) & tvec_{3},\\ 0 & 1 \end{array}\right]\\ H_{2\to 1} & =H_{1}^{-1}\cdot H_{2}, & \;H_{3\to 1} & =H_{1}^{-1}\cdot H_{3}. \end{array}$$

### 2.2. Tasks and Subjects

The tasks were based on the “Sea Spikes Model”, which is available in the da Vinci Skill Simulator and as a real model (see Figure 1) [26]. It offers an easy entry-level task with the da Vinci, yet still requires (and develops) various important skills such as precision, concentration, ambidexterity, and force modulation.

For the whole experiment, a da Vinci XI with two large needle drivers was used. The same master console with the da Vinci Skill Simulator was employed for the simulated tasks. In the first trial, there were three groups of subjects: medical students, resident surgeons, and laparoscopic surgeons before their board certification, with five subjects in each group. Before the sessions, various potentially relevant metrics were recorded by questionnaires, such as number of completed laparoscopic surgeries, initial fatigue, initial pulse, sports habits, etc.

The sea spike tasks included the multicolored sea spikes model (about 10 cm diameter), which is made of soft silicon, and rubber rings (about 5 mm diameter) placed close to it with the same set of colors. The rings should be placed on the spikes with matching colors one by one using two forceps, with a maximum of ten rings on the ten spikes. Dropping rings, missing colors, and instrument collision are the most common errors; however, the simulator measures movement effectiveness as well. The exact tasks of this experiment were as follows:

1. Place all ten rings on the spikes*
2. Place as many rings on the spikes as possible within 2 min
3. Place as many rings on the spikes as possible within 2 min under disturbance
4. Simulator: Place as many rings on the spikes as possible within 2 min**

* During task one, subjects had a 20-minute free practice session after the first exercise. ** An extra task for novices was an additional simulator exercise: “Place all rings on the spikes”. The fifth task for novices was the same as the fourth task for the surgeons the residents; later, this extra task was excluded from the study.

### 2.3. Classification and Parameter Tuning

Most recent research activities have employed artificial intelligence-based methods for surgical skill assessment and classification; thus, machine learning algorithms were utilized in this part of the study [12,27]. The dataset (HR, hand movements, posture) was collected with different devices and comprised eleven features: one from HR, six from hand movements (right- and left-hand *x, y, z* coordinates), and four from posture (right shoulder and elbow *x, y* coordinates). After each trial, the participants filled out the Surgical Task Load Index (SURG-TLX) [28] questionnaire about the mental, physical, and temporal demands, task complexity, situational stress, and distractions. The participants answered each question using scores from 1 to 20. For instance, the scores for mental demand, 1 indicated minimal mental demand while 20 denoted a high level of mental demand. The target variables for classification were derived from these responses.

In order to use the given target variables for binary classification to classify the surgeons into novice and expert groups, it was imperative to transform the scale of the results from a range of 1 to 20 into a binary format. This transformation was accomplished by applying a simple condition to each of the six responses. When the response was lower than the mean value of the responses, the variable was transformed into a 0, representing the expert class. Conversely, when the response exceeded the mean value it was assigned a value of 1, indicating the novice class.

For the purpose of classification, Decision Tree (DT), k-Nearest Neighbors (k-NN), Support Vector Machine (SVM), and Logistic Regression (LR) were used as non-time series classifiers [29,30]. These classifiers are unsuitable for the direct utilization of time series data as input. To convert the kinematic features, which inherently exhibit a time series structure, into a format that was suitable for the classifiers, the Approximate Entropy (ApEn) was employed from the entropy library [31].

To achieve greater accuracy, the method of parameter tuning was implemented through GridSearchCV in order to fine-tune the classifier parameters and identify the best training and test sets using various cross-validation methods. The results were achieved using two different validation methods. The first method involved Leave-One-Out Cross-Validation (LOOCV), wherein a single trial was separated for testing during each iteration, with the remaining trials were used for training. The second approach was k-fold cross-validation, where k ranged from 2 to the maximum possible fold number. This method first separates the input data into a specified number (k) of folds. One trial from each fold was used for testing and the rest for training.

The tuned DT parameters were the criterion, the function employed to quantify the quality of a split (‘gini’, ‘entropy’); max_depth, the maximum depth of the tree (None, range from 1 to 10); max_features, the maximum number of features considered for each split (None, ‘sqrt’, ‘log2’, ranging from 0.1 to 1); and the splitter, the strategy employed for partitioning each node (‘best’, ‘random’). For k-NN and SVM, only three parameters were tuned. The k-NN parameters were: n_neighbors, the maximum number of neighbors (ranging from 1 to the maximum possible neighbors); weights, the process of assigning weights to neighboring data points (‘uniform’, ‘distance’); and metrics, the metric used for distance computation (‘euclidean’, ‘manhattan’, ‘minkowski’). For SVM, we tuned the following parameters: C, the regularization parameter, which is a trade-off between the maximization of the margin and the minimization of the misclassifications (1000, 500, 100, 50, 10, 5, 1, 0.5, 0.1); kernel (‘poly’, ‘rbf’, ‘sigmoid’, ’linear’); and gamma, the influence of a single training example (‘auto’, ‘scale’, 1, 0.5, 0.1, 0.05, 0.01, 0.005, 0.001, 0.0005, 0.0001).

LR was different from the other classifiers, as tuning the values of the three parameters can only be employed if the solver can use the penalty. Notably, not all solvers can use all of the penalty types; consequently, the Python code used for classification paired only the usable penalties for a specified solver. The solver parameter was tuned using ‘newton-cg’, ‘lbfgs’, ‘liblinear’, ‘sag’, and ‘saga’, and the values for the penalties were ‘l1’ and ‘l2’. The third parameter (C, the inverse of the regularization strength) involved testing different values such as 0.1, 0.5, 1, 5, 10, 50, 100, 500, 1000.

## 3. Results

The most important outcome of the presented initial experiment was to set up a complete methodology (tasks, measured metrics, measurement methods, etc.) and a practical pipeline for data collection that can be used in the long term for stress and ergonomics analysis in robot-assisted surgery. In this section, we present our findings based on the analysis of the collected initial dataset regarding stress-related metrics and skill level classification. The experience gained, lessons learned, and planned modifications for future measurements are presented in Section 4.

**Figure 3:**
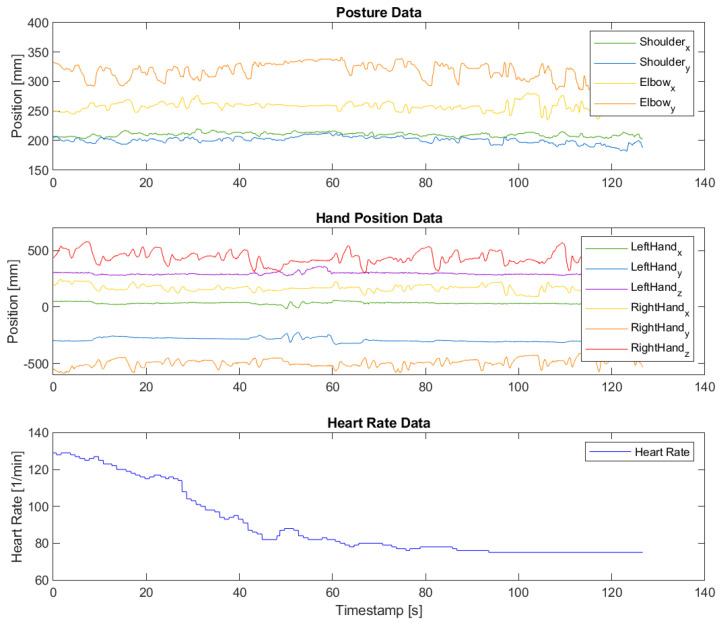
Synchronized posture, MTM-tracking, and HR data of a typical subject. It can be seen that the subject barely used his left hand and that his heart rate relaxed during the trial.

The first step of data analysis was synchronizing measurement data from the different sensors. First, the HR data (recorded directly into Polar’s cloud storage) was synchronized with the MTM tracking data, as both were recorded with UNIX timestamps. Posture detection was performed offline on the recorded videos; thus, those datasets were synchronized to the others manually based on the videos. Figure 3 shows some typical synchronized graphs.

### 3.1. Correlations

The main goal of the analysis of the initial measurement data was to determine and validate applicable methods for estimating stress level and to find other correlated metrics. The stress level of the subjects was estimated using the Baevsky Stress Index, calculated from the RR-intervals output of the Polar H5 heart rate monitor for each task of each subject.

The calculated Pearson Correlation Coefficients (PCC) are shown in Table 1 along with the *p*-values indicating the significance of the hypothesized correlations (either negative or positive). The examined data included kinematic metrics derived from hand position tracking (e.g., the bounding box of hand movements, average speed, jerk, etc.), the ratios of these metrics between the two hands, posture metrics (vertical displacement of the arms), the SURG-TLX questionnaire answers, and manually recorded scores such as the number of collisions (either between the two forceps or between the forceps and the sea spikes model), ring drops, and successful ring placements. Strong statistically significant correlation was concluded where the *p*-value was below 0.05, while weak or possible correlation can be assumed where the *p*-value is between 0.05 and 0.08.

Strong positive correlation was observable between BSI and two left-hand movement metrics, namely, the 3D spatial standard deviation and the range, i.e., the size of the bounding box of the left-hand movements. These two metrics have similar meanings, with both being related to the spatial extent of the hand movements, which is often correlated with the level of experience [32,33]. On the other hand, the total path covered, average speed of the left hand, and hand usage rate metrics did not show any correlation, leading to the assumption that increased stress levels result in wider movements with the non-dominant hand (all subjects were right-handed) but not better bimanuality. Furthermore, the total distance covered with the right (dominant) hand showed a strong negative correlation with the stress level, indicating that the subjects tended to be more effective under this amount of stress. The bimanuality-related metrics (distance covered and movement range ratios) showed weak negative correlations, indicating that increased stress worsened bimanuality even further (the average ratio for the whole dataset between dominant and non-dominant hand usage was 1.7, and the ratio of bounding box sizes was 6.2).

Among the self-assessed SURG-TLX metrics, two significant correlations were found. The data shows that BSI was correlated with self-reported physical fatigue, but not with mental fatigue. This could be caused by different interpretations of the two metrics, as subjects tended to give much higher values for mental fatigue (avg. 6.6/10) than for physical fatigue (avg. 2.8/10), meaning that increase can be detected more easily for the latter. The fact that the “Situational Stress” metric showed no correlation indicates that the stress estimation method was unreliable or that the subjects did not understand the question or scale. Distraction, on the other hand, produced the expected correlation, meaning that the distractions used during the third task delivered their anticipated stress-increasing effect. Among the manually recorded scores and mistakes, only one weak negative correlation was observable, which was between the number of ring drops and BSI; thus, this level of stress did not have a direct influence on performance.

### 3.2. Classification by Supervised Learning

The results of the classification, including parameter tuning, are presented in Table 2. Each row in the table represents the accuracy for one of the six questions from the SURG-TLX questionnaire. The best accuracy scores are highlighted in bold. Notably, the table also shows the employed cross-validation type. It is not uncommon for a classification algorithm to have the same accuracy across different validation methods; there was one case for situational stress in this dataset in which the k-NN algorithm had the same result with both 13-fold and 15-fold cross-validation. Among the six target variables, the temporal demand (the amount of time pressure associated with completing the trials) yielded the highest result with the Decision Tree algorithm and nine-fold cross-validation. Therefore, using this target variable for classification proved to be the most effective way to separate the surgeons into novice and expert groups.

## 4. Conclusions

This study was driven by the aim of establishing a methodology for assessing stress and ergonomics in RAMIS, paving the way for further research in the field of clinical situational awareness and attention computing. The correlations that we found between stress levels and kinematic metrics along with the potential of machine learning for skill level classification offer a valuable base for further wider-ranging research activities with the potential to optimize surgical training and potentially improve patient outcomes. Future research should focus on expanding the dataset, refining measurement methods, and exploring the implications of these findings for RAMIS practice and training.

### 4.1. Lessons Learned

Although the presented first set of measurements was completed mostly successfully, some important flaws, smaller mistakes, and unnecessary steps were clearly identified. In general, the selection and design of the tasks are the most important part of such experiments; they have to be practically aimed at the exact research question(s) while being reproducible, and should require skills with as many objectively measurable metrics and outcomes as possible while also not being too difficult. It was useful to offer a 20–30 min uncontrolled practice session during which learning curves were not examined, which allowed inexperienced participants to grow accustomed to the new environment and controls.

In the case of posture detection, the attire worn by the participants holds significance, as the presence of a striped t-shirt or sweater in the video introduces limitations in accurately identifying the body landmarks with the used Python code. Another critical concern involves the participants’ head position at the beginning of the video. The subjects should directly look into the camera positioned on their right sides, otherwise the code will fail to recognize the human body, leading to the absence of all points.

In light of the inherent subjectivity of SURG-TLX metrics, the risk of participants underestimating their stress levels is present. Subjects might either consciously or subconsciously try to present themselves in a better light, or may lack self-awareness regarding their own mental fatigue level. This phenomenon, known as “response bias”, further underscores the importance of integrating objective measures for estimating stress. Furthermore, the lack of the expected correlation between “situational stress” and BSI indicates that more detailed explanation of the SURG-TLX metrics is needed. To tackle the undesired effect of response bias, there was agreement that the scale of the SURG-TLX questionnaire should be explained by separate phrases for each question, allowing the participants to interpret the scales similarly. To obtain more reliable stress level estimations, it would be possible employ eye-tracking glasses in future measurements, as they offer stress level estimation calculated from pupil tracking metrics [34]. These findings might be beneficial for similar future works by contributing to the mitigation of inaccuracies in measurements and data generation.

### 4.2. Future Work

The created dataset contained a relatively small amount of data (58 trials), and data from professional RAMIS surgeons have not yet been recorded and included in the analyses. Including such data would enhance the skill level classification and make the stress-related data more diverse.

To make the training dataset more efficient and suitable for classification, more data are required from more surgeons with varying levels of surgical experience. Several methods can be employed to increase the best possible accuracy, such as the standardization method, as some of the non-time series algorithms (especially the k-NN classifier) could achieve higher results with a standardized dataset. Beyond these methods, other types of classifier implementations, particularly time series classifiers such as Neural Network or Dynamic Time Warping, could enhance the achieved results. These implementations could even provide a comparative analysis between the two classifier types.

A new data collection system consisting of a head-mounted eye tracking device could be introduced to enhance the stress level estimation based on pupil metrics as additional input data. In addition, the resting heart rate should be recorded (including retrospectively for the first group as well).

It is believed that the recognition and maintenance of clinical situational awareness will lead to the earlier discovery and alleviation of clinical adverse events.

## Figures and Tables

**Table 1:** Pearson correlations between the Baevsky Stress Index values and other measured and calculated metrics. Correlations with *p*-values < 0.05 are considered to be strong and significant (bold rows), while those with 0.08 < *p* < 0.05 show possible correlations worth examining with larger datasets. The ‘+’ and ’−’ signs indicate positive and negative correlations.

Metric Group	Metric	Pearson Correlation	*p*-Value	Significance
Hand movement metrics	**Lefthand_std**	**0.370**	**0.006**	**strong +**
	**Lefthand_range**	**0.288**	**0.037**	**strong +**
	Lefthand_total_dist	0.062	0.658	-
	Lefthand_avg_speed	0.173	0.216	-
	Left_jerk	0.065	0.641	-
	Righthand_std	0.164	0.242	-
	Righthand_range	0.042	0.767	-
	**Righthand_total_dist**	**−0.364**	**0.007**	**strong −**
	Righthand_avg_speed	−0.135	0.335	-
	Right_jerk	0.001	0.993	-
	Total_dist_rate	−0.251	0.069	weak −
	Range_rate	−0.256	0.064	weak −
Posture metrics	Elbow_vy	0.062	0.661	-
	Shoulder_vy	−0.014	0.921	-
SURG-TLX metrics	Mental_fatigue	0.198	0.172	-
	**Physical_fatigue**	**0.380**	**0.007**	**strong +**
	Temporal_demands	0.084	0.567	-
	Complexity	0.223	0.124	-
	Situational_stress	0.082	0.577	-
	**Distractions**	**0.284**	**0.048**	**strong +**
Manually recorded metrics	Collisions_phantom	−0.124	0.396	-
	Collision_robot_arms	−0.205	0.158	-
	Rings_placed	0.194	0.182	-
	Ring_drops	−0.269	0.061	weak −
	Spike_color_missed	−0.054	0.714	-

**Table 2:** Accuracy achieved for the different target variables. The highest accuracy for each variable is represented in bold. By tuning its parameters, the Decision Tree classifier reached the highest accuracy when using temporal demand as the predictive variable for binary classification. Abbreviations: DT: Decision Tree, k-NN: k-Nearest Neighbors, SVM: Support Vector Machines, LR: Logistic Regression, MF: Mental Fatigue of the task, PF: Physical Fatigue of the task, TD: Temporal Demand, C: Complexity of the task, SS: Situational Stress, D: Distractions, cv: number of cross-validation folds, LOOCV: Leave-One-Out Cross-Validation.

Target Variable	DT	k-NN	SVM	LR
MF	cv = 11: **0.781818**	cv = 18: 0.712963	cv = 7: 0.734694	cv = 15: 0.638889
PF	cv = 20: 0.758333	LOOCV: 0.693878	cv = 9: **0.807407**	cv = 17: 0.754902
TD	cv = 9: **0.818519**	cv = 8: 0.758929	cv = 8: 0.693452	cv = 15: 0.733333
C	LOOCV: **0.734694**	cv = 13: 0.621795	cv = 23: 0.688406	cv = 20: 0.583333
SS	cv = 21: 0.761905	cv = 13, 15: 0.666667	cv = 17: **0.784314**	cv = 6: 0.574074
D	LOOCV: **0.734694**	cv = 17: 0.676471	cv = 6: 0.712963	cv = 16: 0.697917

## Data Availability

The raw data supporting the conclusions of this article will be made available by the authors on request.
